# COVID-19 Vaccine-Induced Rapid Progression of Prediabetes to Ketosis-Prone Diabetes Mellitus in an Elderly Male

**DOI:** 10.7759/cureus.28830

**Published:** 2022-09-06

**Authors:** Binay Kshetree, Janette Lee, Sameer Acharya

**Affiliations:** 1 Internal Medicine, Cayuga Medical Center, Ithaca, USA

**Keywords:** diabetes mellitus in elderly, covid19 vaccine side effects, covid-19 vaccine complication, prediabetes, dysglycemia, diabetes type 2, type i diabetes mellitus, covid 19

## Abstract

Studies show a low progression rate of prediabetes to Type 2 diabetes mellitus (DM) that we commonly seek to reverse, but we don’t associate prediabetes as a lead-up to the first presentation of ketosis. We present a prediabetic who, in less than a year, converted to GAD65 antibody-positive diabetes mellitus with a diabetic ketoacidosis presentation. A 69-year-old male presented with three weeks of fatigue, polyuria, polydipsia, abdominal pain, and weight loss. Vital signs and physical exam were normal except for abdominal tenderness and dry oral mucosa. Complete blood count (CBC) was normal; blood glucose was severely elevated with mild corrected hyponatremia; elevated anion gap metabolic acidosis with glucosuria and ketonuria. He received an insulin drip, normal saline, and potassium in the intensive care unit. His anion gap closed overnight and was switched to basal-bolus insulin. Hemoglobin A1c (HbA1c) came out to be higher than expected as compared to last year of low prediabetic value, decreased c-peptide levels, and positive anti-GAD65 antibody. His first abnormal HbA1c was 5.8% a year ago and no autoimmune marker was checked before. He was vaccinated with the messenger ribonucleic acid (mRNA) coronavirus disease 2019 (COVID-19) vaccine a year ago with an mRNA vaccine booster two months earlier. He was not COVID-19 infected. We discharged him with a basal-bolus insulin regimen. Type I DM passes from autoimmunity-positive normoglycemia to dysglycemia to the symptomatic stage, typically progressing more rapidly in children than in older adults. A new Type I or dysglycemia in Type II DM is increasingly reported after COVID-19 vaccines/infection. Mechanisms could be cytokine-mediated beta-cell damage or autoimmunity after mRNA vaccines or as a part of autoimmune syndrome induced by vaccine adjuvants. This case reports the rapid progression of prediabetes to Type 1 rather than Type 2 DM and highlights the possibility of dysglycemia after COVID-19 vaccines and calls for measures to prevent or early management of these side effects.

## Introduction

While a 5-10% rate of progression from prediabetes to Type 2 diabetes mellitus (DM) is a familiar phenomenon, and one that we seek to reverse, we rarely associate prediabetes as a lead-up to the first presentation of Type 1 DM with diabetic ketoacidosis [1]. Type 1 diabetes mellitus of adult-onset is rare, to begin with, but those who develop it typically start as Type 2 and become Type 1 after a prolonged, progressive beta-cell failure [2]. Reports of quick progression within a year of prediabetes are exceedingly rare. Here, we present a prediabetic who within a year converted to anti-GAD65 antibody-positive Type 1 DM with a recent history of COVID-19 vaccination that could have been associated with its rapid progression.

## Case presentation

A 69-year-old male with a past medical history of hypertension and prediabetes presented with worsening fatigue, polyuria, polydipsia, blurry vision, and 14 pounds of weight loss over three weeks. He also reported intermittent upper abdominal pain and episodic ambulatory lightheadedness for two weeks. He also reported a loss of taste and smell for two weeks with a diminished appetite. His home medications were irbesartan and chlorthalidone for hypertension; he also used to take aspirin intermittently. His family history was significant for some members with graves disease and celiac disease. He was a non-smoker with no alcohol or illicit drug use. On examination, he was afebrile with normal blood pressure, sinus tachycardia, and mild tachypnea. Physical exam was remarkable for mild upper abdominal tenderness and dry oral mucosa.

Complete blood count was within normal limits; blood glucose was 750 mg/dl with serum sodium of 123 mmol/l (corrected sodium 133) and potassium 4.4 mmol/l; elevated anion gap of 29 with bicarbonate of 20 mmol/l; blood gas with pH 7.31; urine with glucosuria and 1+ ketonuria; and elevated creatinine (Table 1). He was admitted to the intensive care unit for diabetic ketoacidosis and prerenal acute kidney injury management and was placed on an insulin drip, intravenous normal saline, and potassium repletion [3]. Anion gap closed overnight and subsequently, and he was switched to basal-bolus insulin. His creatinine normalized with fluid repletion. His sodium level also improved with normal saline.

**Table 1:** Laboratory values Lab Abbreviations: *CBC: complete blood count; WBC: white blood cells; MCV: mean cell volume; BUN: blood urea nitrogen; AST: aspartate transaminase; ALT: alanine transaminase; ALP: alkaline phosphatase; LDL: low-density lipoprotein; HDL: high-density lipoprotein; SARS-CoV-2: severe acute respiratory syndrome-coronavirus-2; RSV: respiratory syncytial virus; NA: not available* DM: diabetes mellitus; GAD: glutamic acid decarboxylase; COVID-19: coronavirus disease-19; PCR: polymerase chain reaction; ASIA: autoimmune syndrome induced by vaccine adjuvants; mRNA: messenger RNA

Laboratory tests	Admission	Day 2	Day 3	Discharge
CBC (units)				
WBC (10^3 ^/µL)	9.6	9.7	7.5	7.2
Hemoglobin (g/dl)	15.7	13	13	12.4
Hematocrit (%)	45	36	36	35
MCV (fL)	94	91	91	91
Platelets (10^3 ^/µL)	302	262	207	188
Serum Chemistries (units)				
Sodium (mmol/L)	123	131	135	132
Potassium (mmol/L)	3.4	3.5	3.1	3.4
Chloride (mmol/L)	74	91	95	96
Bicarbonate (mmol/L)	20	26	31	32
Anion Gap (mmol/L)	29,22	13,5	6	4
BUN (mg/dL)	36,34	31,29	21	18
Creatinine (mg/dL)	1.44,1.32	1.17	0.87	0.91
Calcium (mg/dL)	10.3	9.5	9.5	9
Magnesium (mg/dL)	NA	NA	2.2	2
Glucose (mg/dL)	750, 532	333,220	111	164
Hemoglobin A1C (%)	13.7			
C reactive protein (mg/dL)	1.67			
Liver function test (units)				
AST (U/L)	16			
ALT (U/L)	20			
ALP (U/L)	55			
Bilirubin (mg/dl)	0.9			
Lipid panel (units)				
Total cholesterol (mg/dL)	184			
LDL cholesterol (mg/dL)	105			
HDL cholesterol (mg/dL)	40.4			
Triglycerides (mg/dL)	193			
Others (units)				
Insulin level (mcIU/ml) (2-16)		78.4		
C peptide level (ng/mL) (1.1-4.4)		0.4		
Anti-GAD65 antibody (nmol/L) (<=0.02)		0.33		

Urine analysis	Admission
Urine pH	5
Urine specific gravity	1.015
Urine protein	Negative
Urine ketones	1+
Urine blood/nitrate/bilirubin/leukocyte esterase	Negative
Urine WBC	Trace
Urine glucose	3+

Nasopharyngeal PCR	Admission
SARS-CoV-2	Negative
Flu A/B	Negative
RSV	Negative

Blood Culture, Aerobic/Anaerobic	No Growth
Urine Culture	No Growth

Hemoglobin A1C trend	2 years ago	1 year ago	Admission	Follow up
Results %	5.1	5.8	13.7	10.3

Lab workup revealed hemoglobin A1c (HbA1c) of 13.7%, decreased c-peptide levels of 0.4 ng/ml (1.1-4.4) and elevated anti-GAD65 antibody level of 0.33 nmol/l (normal < 0.02). He never had autoimmune antibody checked prior to this admission as he was prediabetic with his last HbA1c 5.8% one year previously and was normal before. No infections were found with negative blood and urine cultures. He was transferred to the general floor on hospital day two after stabilization. His blood glucose was labile with daytime and nighttime excursions that required optimization of insulin dosing. On hospital day three, he reported persistent fatigue, which improved with the optimization of blood glucose by adjusting the dose of long-acting glargine and short-acting lispro insulin.

He was vaccinated with the messenger ribonucleic acid (mRNA) COVID-19 vaccine one year back, receiving his mRNA booster two months earlier. COVID-19 polymerase chain reaction (PCR) test was negative for this admission. He denied any history of COVID-19 infection in the past. His low-density lipoprotein (LDL) cholesterol was 104 mg/dl and triglycerides were 193 mg/dl.

He was given diabetic education about new insulin-dependent diabetes mellitus and was discharged from the hospital with a basal-bolus insulin regimen optimized with a moderate-intensity statin. On a three-week endocrinology follow-up, his repeat HbA1c improved to 10.3%. He continues to be insulin dependent and regularly follows up with his physician.

## Discussion

The era of COVID-19 and its global effects as a pandemic is apparent now. There have been exciting developments in the mRNA and DNA vaccines against this virus that have proven beneficial effects on immunity and preventing transmission. However, we author here wanted to show the endocrine effects of the COVID-19 vaccine. Type 1 DM passes through stages from autoimmunity-positive normoglycemia to dysglycemia to symptomatic Type 1 DM, which typically progresses much more rapidly in younger children than in older adults [2]. We are reporting this case as a rare association between the COVID-19 mRNA vaccine and DM progression which can act as exciting inclusion to the potential prospective studies. A family history of autoimmune disease may have predisposed him to develop diabetes mellitus with Type 1 features. But, this rapid progression, especially within a year of detection of low prediabetic HbA1c to severely high HbA1c with ketoacidosis at presentation preceded by a recent COVID-19 vaccine administration, shows a temporal association and probable cause of this endocrine exacerbation. Dysglycemia in Type 1 or 2 DM is increasingly reported after COVID-19 infection or vaccination but rarely reported about the rapid progression of prediabetes to Type 1 diabetes mellitus [4]. Postulated mechanisms are cytokine-mediated pancreatic beta-cell damage or autoimmunity after mRNA vaccines or as a part of autoimmune syndrome induced by vaccine adjuvants (ASIA syndrome) [5-7]. Although his autoimmune antibodies were never checked before this ketoacidosis admission due to his asymptomatic low prediabetic state, we can associate rapid emergence or exacerbation of autoimmune antibodies after the administration of COVID-19 vaccines given the patient received vaccine one year back with a recent booster two months ago leading to ketosis prone symptomatic DM in this case.

## Conclusions

COVID-19 infection and vaccines have been reported to be associated with various organ-specific complications. We want to show the importance of preventive screening and timely management of dysglycemia that could present as symptomatic hyperglycemia after COVID-19 vaccination. With reports of the associated endocrine effects of these vaccines, especially on the pancreatic endocrine cells, the identification of various other autoimmune diseases and post-vaccination syndrome shouldn’t be overlooked and would help with the proper identification of unique health issues a patient may bring in this post COVID-19 era where vaccination is always recommended.

## References

[1] Tabák AG, Herder C, Rathmann W, Brunner EJ, Kivimäki M (2012). Prediabetes: a high-risk state for diabetes development. Lancet.

[2] Insel RA, Dunne JL, Atkinson MA (2015). Staging presymptomatic type 1 diabetes: a scientific statement of JDRF, the Endocrine Society, and the American Diabetes Association. Diabetes Care.

[3] Dhaliwal R, Weinstock RS (2014). Management of type 1 diabetes in older adults. Diabetes Spectr.

[4] Aberer F, Moser O, Aziz F (2022). Impact of COVID-19 vaccination on glycemia in individuals with type 1 and type 2 diabetes: substudy of the COVAC-DM study. Diabetes Care.

[5] Lim S, Bae JH, Kwon HS, Nauck MA (2021). COVID-19 and diabetes mellitus: from pathophysiology to clinical management. Nat Rev Endocrinol.

[6] Sasaki H, Itoh A, Watanabe Y (2022). Newly developed type 1 diabetes after coronavirus disease 2019 vaccination: a case report. J Diabetes Investig.

[7] Shirakawa J (2021). Pancreatic β-cell fate in subjects with COVID-19. J Diabetes Investig.

